# Irisin and its relationship with metabolic health: more questions than answers

**DOI:** 10.20945/2359-3997000000355

**Published:** 2021-04-05

**Affiliations:** 1 Hospital 9 de Julho Centro de Controle de Peso São Paulo SP Brasil Centro de Controle de Peso, Hospital 9 de Julho, São Paulo, SP, Brasil

Irisin is a proliferator-activated receptor-gamma coactivator-1α (PGC-1α)-dependent myokine. It was identified in 2012 and, at that time, received a lot of attention due to the hypothesis that drives the browning of white adipose tissue, leading to metabolic benefits and increased thermogenesis (1,2). The original research suggested that irisin is secreted after cleavage of the extracellular domain of a precursor transmembrane protein called fibronectin type III domain-containing protein 5 (FNDC5) in response to exercise (1). As such, irisin could be an important effector of the metabolic effects of physical activity in human health and a potential therapeutic target for diseases, such as obesity and type 2 diabetes (2). Nevertheless, further studies have questioned the relevance or even the existence of irisin in humans (3,4). These issues have arisen due to several reasons, as the start codon (ATA) of the human FNDC5 (which is different from the canonical start codon ATG, present in other mammals) is generally associated with very low protein synthesis. Besides, there are claims that many of the commercially available assays for irisin detection lack specificity (3,4). Moreover, there is no evidence, in the available assays, that irisin is secreted in humans after regular physical activity practice, or that it has any effect on browning, in the available assays (3) and most trials shows that its levels are generally increased in obesity and metabolic syndrome (2,3,5–7).

In this issue of the *Archives of Endocrinology and Metabolism*, two articles show new data concerning the controversial field of irisin levels in humans and add more pieces to the puzzle, although we clearly still have more questions than answers (8,9).

Werida and cols. evaluated 176 subjects and randomized them by BMI status and gender (8). In agreement to several other studies, they observed irisin as positively associated with BMI and HOMA-IR in men and women, with higher levels detected in men.

What is the possible significance of elevated irisin in obesity and its positive correlation to insulin resistance? One proposal is the existence of “irisin resistance”, similar to what happens to leptin (interestingly, irisin is also secreted in low levels by the adipose tissue) or, alternatively, the myokine secretion could be an adaptive response in order to curb the detrimental metabolic consequences of weight gain (2,3,7).

On the other hand, weight gain and insulin resistance are associated with whole-body (and muscle) oxidative stress. Thus, it is possible, as previously discussed in an editorial at this same Journal, that irisin could be a marker of chronic oxidative stress secreted after muscle injury (10). The results of the study by Werida and cols. point in this direction as well. Why? In this research, plasma irisin levels were closely correlated to clusterin and IL-6, two additional controversial markers of metabolic health.

Clusterin, also known as ApoJ, is a molecular chaperone, which could have an important role in the clearance of cellular debris. Clusterin levels, however, are generally elevated in situations of increased chronic inflammation and oxidative stress, and a raise in its expression is associated with worsened liver fat in animal models (11,12). IL-6, which is an adipokine and a myokine, has also been associated with poorer metabolic health. Nonetheless, similarly to irisin, some trials have shown metabolic benefits of its infusion, leading to decreased energy intake and improvement in insulin sensitivity (13).

This major correlation between these “three musketeers” strongly suggests that irisin could be, as both other molecules, a marker of oxidative stress, thereby, decreasing the likelihood of being physiologically important for the beneficial effects of physical activity.

As a potential target to treat metabolic diseases, it is not surprising that the role of irisin in blood pressure has been investigated, although much less than its effects on body weight and glycemia (7,9,14,15). In a rodent model, central irisin infusion was associated with increased blood pressure, although peripheral infusion has reduced it (16). Its peripheral effects are postulated to be mediated by reducing endothelial dysfunction (15,16). In humans, as irisin is positively associated with metabolic syndrome, it also correlates positively with systolic and diastolic blood pressure in most (but not all) studies (7,9,14,15).

Accordingly, Miazgowski and cols. evaluated the role of irisin in young adults with hypertension (9). Since there is no control group, their aim was to investigate whether irisin levels were correlated to the severity of hypertension or dipping (nocturnal systolic blood pressure fall) in already hypertensive individuals. There were no correlations of irisin levels to any of the evaluated parameters. Although they concluded that plasma irisin levels were normal, we must keep in mind that irisin reference values were not validated to date (3), so we should alternatively conclude that in a cluster of young individuals with hypertension, irisin levels is not associated with the severity of the disease.

Nevertheless, the observations offered by both trials do not mean that pharmacological levels of irisin could not be potential therapeutic targets for metabolic diseases in the future, as its infusion leads to reduced insulin resistance, increased thermogenesis, and reduced blood pressure, at least in rodent models (2,3).

As an example, almost 50 years after the discovery of incretin hormones, there is still a debate going on whether obesity and diabetes are related to diminished incretin secretion and action, and GIP (glucose-dependent insulinotropic polypeptide) is possibly physiologically more relevant than GLP-1 (glucagon-like peptide-1) in its incretin effects (17). Nonetheless, GLP-1 agonists have evolved as a class of drugs highly effective in both type 2 diabetes and obesity, probably with independent cardiovascular benefits (18). In this context, although associative studies can bring us insights, rodent genetic knock-out or gain of function models, as well as irisin infusion studies, could offer us more clues and, hopefully, lead to intervention studies in humans in the future.
